# The Current Shortcomings and Future Possibilities of 3D Printed Electrodes

**DOI:** 10.1021/acs.analchem.4c02127

**Published:** 2024-08-28

**Authors:** William B. Veloso, Thiago R. L. C. Paixão, Gabriel N. Meloni

**Affiliations:** Institute of Chemistry, Department of Fundamental Chemistry, University of São Paulo, São Paulo, SP 05508-000, Brazil

## Abstract

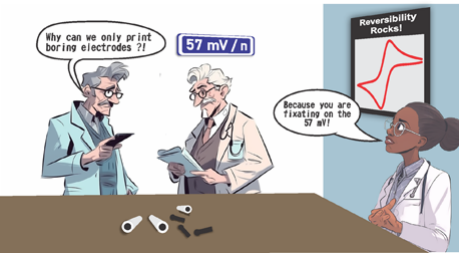

3D printing has changed many industries and research areas, and it is poised to do the same for electrochemistry and electroanalytical sciences. The ability to easily shape electrically conductive parts in complex geometries, something very difficult to do using traditional manufacturing techniques, can now be easily accomplished at home, opening the possibility of fabricating electrodes and electrochemical cells with geometries that were once unimaginable. This ability can be a milestone in electrochemistry, allowing the fabrication of systems tailored to specific applications. Unfortunately, this is not what is seen to date, with 3D printing mostly reproducing “traditional” designs, using little of the “freedom of design” promised by the technology. We reason that these results come from too much focus on reproducing the electrochemical behavior of metallic electrodes instead of understanding how material properties impact the performance of 3D printed electrodes and working within these constraints. 3D printing will change electrochemistry and electroanalytical sciences if we understand and learn to work with its limitations.

## Introduction

The ability to use affordable, desktop-sized 3D printers to fabricate electric conductive materials is shaping the future of electroanalytical chemistry. Once restricted to traditional manufacturing methods (mostly machining) or industrial-grade 3D printing techniques that can sinter metal particles together,^1^ printing conductive parts can now be achieved using fused deposition modeling (FDM) 3D printing at home.^2,3^ The conductivity of FDM printed parts still falls short of metallic conductors, which is easily explained by the nature of the printable material, a mixture of an insulating thermoplastic, usually polylactic acid (PLA), with a conductive additive, usually a carbon allotrope.^4−6^ The carbon conductive surfaces are conducive to performing electrochemical reactions, and unsurprisingly, partially due to the novelty aspect, many deployments of 3D printed electrodes for electrochemical sensing applications have been developed.^7−9^

What might be surprising is the development path that 3D printing electrodes have taken so far and the many shortcomings of the applications of this technology to electrochemistry, which were first introduced 8 years ago.^10^ 3D printing, as a technology, offers incredible freedom to the user, the ability to imagine something and make it materialize within hours and costing very little. This is often themed as “freedom of design” in most manuscripts that use 3D printing for fabricating electrodes. Despite the promises, such design freedom has yet to be delivered in any electrochemical applications. The reasoning behind the shortfalls of 3D printing for electrochemistry, compared to other research fields,^11−13^ might rely on a single aspect that researchers have focused on too much: the reversibility of the voltammetric response of 3D printed electrodes.^14^ Although a key aspect in electrode design and fabrication, the “chase for the reversibility” has guided most efforts toward time-consuming, and sometimes unnecessary,^15^ fabrication processes and severely limited the design of 3D printed electrodes and electrochemical devices.

### Why Reversibility Matters

In electrochemistry, saying a process is reversible means that mass transport is limiting the electrode response, i.e., the rate of the heterogeneous electron transfer (HET) reaction happening at the electrode is not the limiting step; most electrochemical processes involve HET reactions, which have rate constants much larger than the mass transfer rate toward the electrode surface.^16^ For sensing applications, having the largest possible signal (smallest signal-to-noise ratio) is always desirable. For amperometric sensing, this implies the largest reaction rate, that is to say, the largest recorded current. Suppose we forget fluid flow and adsorption or surface-limited processes for now; in this case, the maximum current recorded by an amperometric sensor will be the one limited by the diffusion rate of species toward the electrode, which will be the reversible electrochemical response. That is why most studies involving 3D printed electrodes gauge the reversibility of the fabricated electrodes using the voltammetric response of common redox probes,^14,15^ chasing that 57 mV n^–1^ cathodic–anodic peak separation, where “*n*” is the number of electrons involved in the HET reaction.^17^

### The Problem of Chasing Reversibility

There is none. At least if it is made correctly. The problem is that until recently, most of that chase was naively made, assuming that the HET kinetics (rate constant) limits the voltammetric response of 3D printed electrodes for most redox probes.^14^ The peak separation on a voltammogram is a proxy for HET kinetics but also reports on the ohmic potential loss in the electrochemical systems.^18^ This simple aspect has been overseen by many studies, which calculated HET rate constants using Nicholson’s method regardless of its limitations.^18^ These chases led to a plethora of pretreatment and modification strategies, which resulted in marginal gains in peak separation, still orders of magnitude larger than 57 mV n^–1^, even after extensive surface treatments.^15^

**Figure 1:**
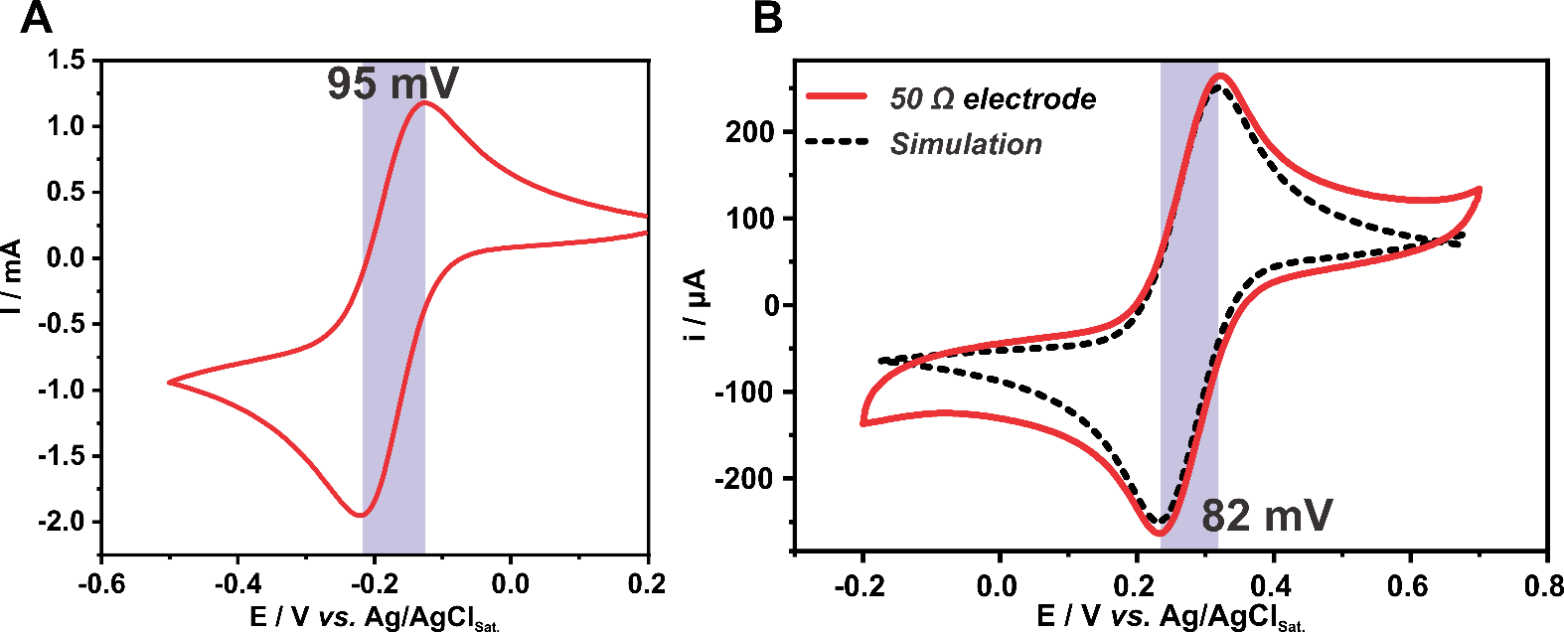
(A) Cyclic voltammograms of 3D printed electrodes in (A) 5 mmol L^–1^ hexaammineruthenium(III) chloride + in 1 mol L^–1^ KCl solution and (B) 1:1 5 mmol L^–1^ [Fe(CN)_6_]^4–^/[Fe(CN)_6_]^3–^ mixture in 1 mol L^–1^ KCl solution (red trace) and numerical simulation of an equivalent geometry electrode with 100% of the surface area electroactive (dashed line). Both voltammograms were recorded at a scan rate of 20 mV s^–1^. Both panels are adapted with permission from ref (14). Copyright 2023 Elsevier.

It is obvious from these results that another aspect limits the reversibility of voltammograms of 3D printed electrodes, and this is the contact resistance arising from the printed material’s electrical conductivity. Contact resistance is a form of ohmic potential loss that will increase peak separation in a voltammogram, and it must be considered when calculating thermodynamic parameters such as the HET rate constant.^18^ This is evident when voltammograms are performed with an outer-sphere redox probe, such as hexaammineruthenium, with a 3D printed electrode (Figure 1A). Ideal outer-sphere species are unsensitive to surface chemistry and have fast HET kinetics.^19−21^ Therefore, the voltammogram, at least in theory, should be reversible, and any peak separation larger than 57 mV (95 mV in Figure 1A) is reporting on contact resistance. The reasoning behind some surface treatment procedures is that the carbon material is dispersed in the polymeric matrix, making the printed electrode a heterogeneous, assessable surface. This would result in large peak separations with the apparent HET rate constant derived from the voltammogram, reporting on the surface coverage of active sites.^22^

This hypothesis falls short by analyzing the voltammogram of the same 3D printed electrode now with its surface covered with metallic gold.^14^ Although another redox species is used here, in this case, the ferri/ferrocyanide redox probe, it is fairly accepted that over metallic surfaces, this probe undergoes a reversible HET reaction.^19−21^ The voltammogram under this condition (Figure 1B) results in a similar potential peak separation (82 mV) to the bare 3D printed electrode. As both cases have fast HET rate constants, and similar contact resistances, the similar peak separation must imply a similar electroactive surface for both electrodes.^22^ When the voltammetric response in Figure 1B is compared to the simulated behavior for an inlaid disk electrode geometry, with an area 100% active and similar contact resistance, a good agreement is seen between peak separation,^14^ signifying that for all cases above (similar peak separations), the entire electrode surface is active or that the interspace between the active sites is smaller than the diffusional length.^22^ The peak separation can be explained only by contact resistance.

### Minimizing Contact Resistance

The problem with the contact resistance of 3D printed electrodes arises from the fact that the printable material has considerable resistivity in its bulk form. The material resistivity might vary from different vendors, storage conditions (particularly humidity)^23^ and age,^24^ and to make matters worse, this resistivity is not translated to printed parts, with an increase in resistivity in the printed electrode material.^25^ Once this was recognized, efforts have been made to understand where in the printing process the bulk conductivity is lost. 3D printing fabricates parts by selectively layering the printable material. The contact and adhesion between each layer can limit the translation of any bulk physical proprieties to the printed part, and it is the reason behind the anisotropy of FDM printed parts, which includes the electrical conductivity.^25^

**Figure 2:**
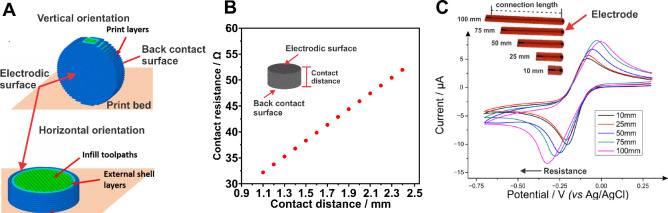
(A) Representation of the orientation of an electrode to be 3D printed, showing it vertically and horizontally orientated. Adapted with permission from ref (28). Copyright 2018 Spring Nature. (B) Effect of contact distance on the contact resistance of 3D printed electrodes, which use back electrical contact. Adapted with permission from ref (14). Copyright 2023 Elsevier. (C) Effect of contact length on the contact resistance, reported by the shift in the peak potential in the voltammograms for 3D printed electrodes. Recorded in 1 mmol L^–1^ hexaammineruthenium(III) chloride + 1 mol L^–1^ KCl solution at 50 mV s^–1^. Adapted with permission from ref (31). Copyright 2022 MDPI.

Patel and co-workers have pioneered the investigation of the impact of some printing parameters on the voltammetric response of 3D printed electrodes. Although there are many printing parameters,^26^ Patel’s group has focused on the effect of printing speed^27^ and orientation of the printed part on the printer bed^28^ on the voltammetric response of 3D printed electrodes. They have shown in both cases that parameters that favor the adhesion between the printed layers or provide a continuous printed path between the electrode surface and the electrical contact toward the potentiostat will result in smaller peak separations. Printing speed on FDM impacts the printed material viscosity with slower speeds resulting in larger residence times of the filament in the heated printer head, leading to lower viscosities and better adhesion and contact between printed layers. This results in lower resistivity of the 3D printed part, lower contact resistance, and lower voltammetric peak separation. The opposite is seen for faster printing speeds.^27^ Printing orientation will be dependent on electrode contact geometry; for back-contacted electrodes, an orientation that favors continuous printed layers between the electrodic surface and the electrical contact surface (vertically orientated for disc electrode – Figure 2A) will decrease contact resistance and improve the reversibility of voltammograms.^28^

Banks et al. have shown that similarly to printing orientation, the layer pattern used to fill the volume of 3D printed electrodes, known as “infill pattern”, can also impact the voltammetric response, with patterns that provide a direct connection between layers favoring the printed part conductivity.^29^ Although, recently, Patel’s group has shown that the infill percentage of a 3D printed electrode bears little on the electrochemical response, with 30% filled electrodes behaving similarly to 100% (solid) 3D printed electrodes.^30^ Banks’s group has also explored the effect of printable material water uptake on the voltammetric response,^23^ showing that the larger the amount of water on the material, the more resistive it is, resulting in larger peak separations in a voltammogram. This points to the fact that properly storing the filament material can help minimize the contact resistance. Water uptake could also be the reason why filament age can impact the voltammetric profile, with older filaments, which had more chance to uptake water, presenting large peak separations.^24^

Contact resistance can also be minimized by the electrode and the electrode contact design.^14^ The connection between a potentiostat and the 3D printed electrode will always be made by a conductive 3D printed path. Given the resistivity of the 3D printed materials, this printed path should be minimized to minimize contact resistance.^31^ Even for back-contacted electrodes (Figure 2B), this is a concern, and the thickness of the 3D printed disc should be kept to a minimum to minimize contact resistance.^14^ For other electrode geometries, the printed connection path should also be minimized to minimize contact resistance and decrease peak separation. This can be done by simply printing a shorter^31^ (Figure 2C) or wider^14^ connection paths.

### Accounting for Contact Resistance

**Figure 3:**
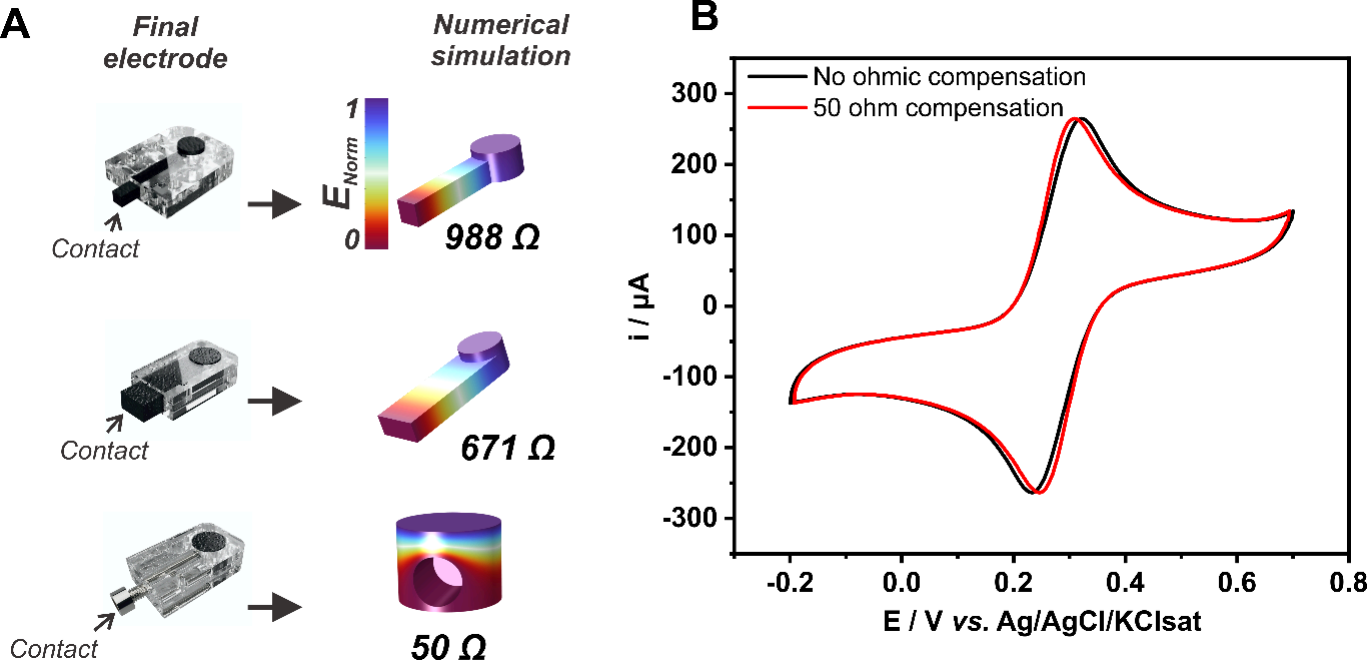
(A) 3D printed electrodes with different contact geometries and the respective numerical simulation for accurately calculate the electrode’s contact resistance. (B) Cyclic voltammograms of the 50 Ω contact resistance electrode recorded in a 1:1 5 mmol L^–1^ [Fe(CN)_6_]^4–^/[Fe(CN)_6_]^3–^ mixture in 1 mol L^–1^ KCl solution at a scan rate of 20 mV s^–1^ without (black trace) and with 50 Ω (red trace) ohmic drop compensation from the potentiostat. Uncompensated Δ*E*_p_ = 82 mV. 50 Ω compensated Δ*E*_p_ = 63 mV. Adapted with permission from ref (14). Copyright 2023 Elsevier.

If we recall the “freedom of design” of 3D printing, electrodes are first precisely drawn to scale using the aid of computer software.^26^ This provides a unique opportunity for understanding the impact of, and accounting for, contact resistance on the voltammetric response of 3D printed electrodes.^14^ Since a virtual model of the electrode exists, the contact resistance of the electrode to be printed can be calculated based on the resistivity of the printed material at a given condition,^25^ and with the use of numerical simulations, this can be done for any electrode/contact geometry, regardless of the complexity (Figure 3A). With the contact resistance value calculated, and knowing the kinetic parameters for the HET reaction that will be used to test the electrode, the voltammetric response of the electrode can be simulated with great accuracy.^14^

There are some important consequences from these; first, knowing the contact resistance value can be used to correct the voltammetric profile in the potentiostat using ohmic drop compensation (Figure 3B). This allows for HET kinetics to be studied with 3D printed electrodes, as once the ohmic drop losses are compensated, peak separation on voltammograms will be reporting HET kinetics,^18^ or surface area coverage for heterogeneous assessable electrodes.^22^ Second, by knowing what the voltammetric profile of the electrode for a diffusion-limited process will be before printing, the development of surface treatments for using these electrodes with inner-sphere redox probes can be guided by chasing the minimal possible peak separation (reversible case) instead of the theoretical 57 mV n^–1^. And third, and possibly more relevant than the other two points, if the voltammetric response can be predicted before printing, it can be optimized for a given application, and tailored for it, which would guide the design of 3D printed electrodes, setting some helpful constraints to the “freedom of design”.

### 3D Printed Electrodes Today

Despite the quick adoption of 3D printing by electroanalytical chemists, most applications do not explore the “freedom of design” promised by 3D printing, with the electrodes and devices printed mimicking commercially available disk electrodes,^30^ or integrated electrode systems, comprising working, pseudo reference and counter electrodes, similar to the also commercially available screen printed electrodes.^32,33^ Although it might be natural to follow what is already known, these designs lack creativity and do not use the capacity to fabricate intricate forms that a literal robot at your service, a 3D printer, can offer. A few 3D printed electrodes^34,35^ are modernized versions of thermoplastic electrodes, which have been used for a long time.^36^ Some 3D printed electrode designs hindered the electrochemical performance of the electrode by essentially creating a cavity electrode.^2^ Some innovative designs have been explored, but are more a novelty in design than applicable.^5,37^

A few attempts have been made on using the capabilities offered by 3D printing to fabricate electrodes in shapes that would be hard to achieve by conventional methods, such as polygonal shaped electrodes,^38^ and printing patterns over the electrodic surface.^39^ A worth noting application that highlights the “freedom of design” of 3D printing is Patel and co-workers faecal pellet shaped electrode for measurements of serotonin and muscle contraction in the intestines of livestock,^40^ or their application of 3D printed electrodes for fabricating microelectrodes,^41^ where ohmic drop losses are (to an extent) unimportant due to the low faradaic currents recorded. These are certainly steps in the direction of using more of what 3D printing can offer, but passed 8 years after the creation of the first 3D printed electrode,^10^ and 5 since the first all-printed electrochemical cell,^2^ it is time for the community to embrace the freedom of design of 3D printing and explore designs that could not be fabricated with other technologies.^26^ 3D printing is a technology to solve problems, not simply a replicating machine.

### The Future of 3D Printed Electrodes

We need to better understand the electrodic surface of 3D printed electrodes, understand the material, its properties, and the impact printing parameters have on it. Although this has been done to an extent, we need more in-depth studies to allow a predictive understanding of the electrochemical performance of any 3D printed electrode design. This could allow for the electrochemical response of a 3D printed electrode to be predicted from the 3D design before printing, helping to optimize the design performance at this stage. We must stop trying to minimize contact resistance and increase reversibility at the cost of electrodes and possibly the entire analytical devices design. If we know the contact resistance and predict it, then we can account for it in the experiments. Stop worrying about it will free the constraints of 3D printed electrode design, allowing complex devices which do not seek to minimize contact length or rely on flat disc shaped geometries, allowing electrodes to be easily embedded in microfluidic channels,^42−44^ hydrodynamic systems,^45^ to be incorporated in sample preparation and solution handling systems,^46−48^ and many others. The integration of an electrode to these systems might require positioning it in a way that the contact path from the electrode to measuring equipment is neither straight nor short. Instead of making such connections externally with wires, they could be printed within the design. These could be fabricated autonomously by a robot that can create bespoke electrochemical devices 24 h a day. Full electroanalytical devices, “Lab-on-a-chip” devices, could be printed in a single machine. The power of such an approach is the decentralization of the production of full analytical devices, which could be produced on demand, where they are needed instead of being shipped to the point-of-need. There is so much more to be done for 3D printed electrodes. Once we realize this, 3D printing will be the revolution in electrochemistry it is promised to be.
